# Whole-heart T_1_-mapping with single breath-hold

**DOI:** 10.1186/1532-429X-17-S1-P389

**Published:** 2015-02-03

**Authors:** Sohae Chung, Pippa Storey, Leon Axel

**Affiliations:** 1 Radiology, NYU Langone Medical Center, New York, NY, USA

## Background

T_1_-mapping, directly measuring the underlying longitudinal relaxation times (T_1_), and extracellular volume (ECV) quantification, are emerging techniques for myocardial fibrosis quantification. Recent studies have reported significant T_1_ differences in fibrotic and normal tissue, but whole-heart T_1_-mapping is rarely performed in clinical practice, due to the associated time-consuming data acquisition; this can lead to sampling error when the fibrotic process is not homogenous. Signal acquisition over multiple heart beats can also be problematic, due to the potential for motion artifacts. In this study, we present a rapid whole-heart T_1_-mapping in a single breath-hold of, e.g., 6 heartbeats (typically, 5-7 seconds for total 9 T_1_ maps).

## Methods

**Figure 1:**
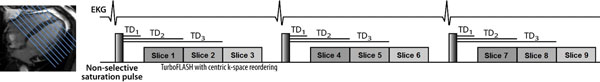
(left) 4CH cine image including 9 imaging locations. (right) Schematic pulse sequence diagram of the whole-heart T_1_-mapping method.

To achieve rapid whole-heart T_1_-mapping, we modified a turboFLASH pulse sequence to acquire multiple T_1_-weighted (T_1_w) images, with increasing sequential time delays (TD), after a non-selective saturation pulse. Whole-heart T_1_-mapping was performed using a 1.5T MRI scanner (Avanto, Siemens). Within three heart beats, we acquired 9 T_1_w images at different levels, with increasing sequential time delays TD=200 (for slices 1,4,7), 397 (for slices 2,5,8) and 594 ms (for slices 3,6,9) after a non-selective saturation pulse (Fig. 1). Centric k-space acquisition ordering is used to minimize the sensitivity to inflow effects and to reduce the sensitivity to B_1_^+^ profile after image normalization. In the first three heartbeats, 9 corresponding proton density-weighted (PDw) images are acquired, in order to correct for the B_1_^-^ and the unknown equilibrium magnetization, and normalize the signal. Post-contrast T_1_ maps were acquired 37 minutes after contrast injection (0.15mmol/kg of gadolinium-DTPA). Using the Bloch equation, T_1_ is obtained from the normalized signal, S^norm^ (=S_T1w_/S_PDw_), and TD: T_1_ = -TD/ln(1-S^norm^).

## Results

**Figure 2:**
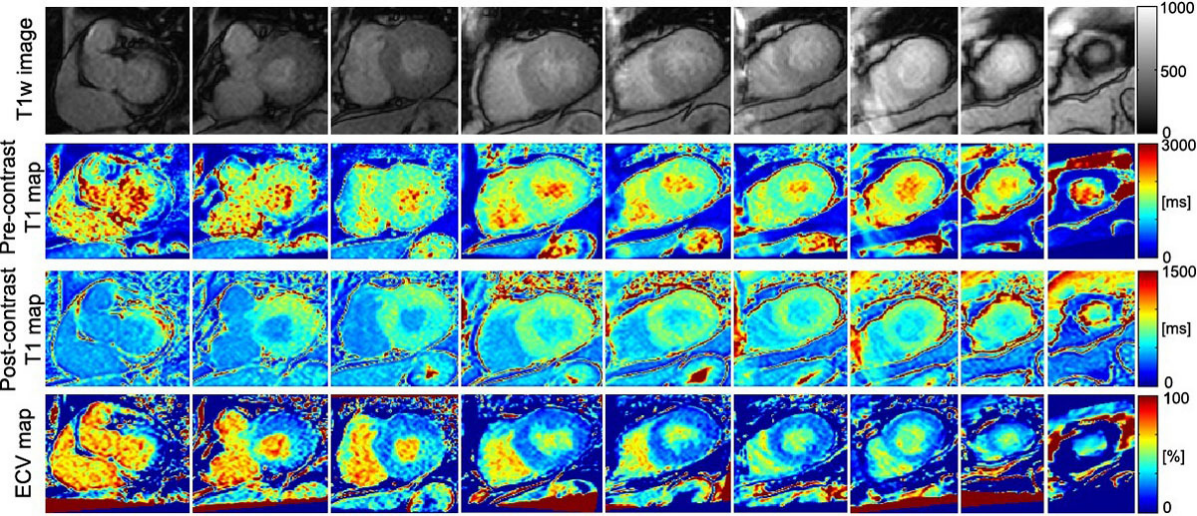
(top) T_1_w images at different levels. (middle) pre- and post-contrast (37 minutes after contrast injection) T_1_ maps. (bottom) ECV maps.

Figure 2 shows the results from a representative patient with hypertrophic obstructive cardiomyopathy (56 years old; male; EF=75%; maximum myocardial thickness=15mm; no focal late gadolinium enhancement (LGE)). Total scan time for this representative patient was 5.4s for 9 slice locations; and pre-contrast myocardial T_1_ values of slice 3-8 were 1421±158ms and ECV values (assuming hematocrit of 0.4) were 0.24±0.05. Although there is no focal LGE, this patient shows a higher pre-contrast T_1_ than in normal controls (T_1_ of ~1s at 1.5T), suggesting a higher degree of diffuse myocardial fibrosis.

## Conclusions

While conventional T_1_-mapping methods are very time-consuming for routine clinical application, this whole-heart T_1_-mapping method can be performed well in patients with cardiac disease-related problems, including arrhythmia or difficulty with breath-holding, due to its short acquisition time of, e.g., 6 heartbeats. Better characterizing whole-heart fibrosis may allow for more accurate and earlier diagnosis. Further studies are warranted.

## Funding

None.

